# Plasma miR-134-5p: a candidate biomarker for predicting non-response to anti-TNF therapy in rheumatoid arthritis

**DOI:** 10.3389/fimmu.2026.1783026

**Published:** 2026-03-17

**Authors:** Arkaitz Mucientes, José Manuel Lisbona-Montañez, Patricia Ruiz-Limón, Rocío Bautista, Diego Lozano-Peral, Sara Manrique-Arija, Aimara García-Studer, Fernando Ortiz-Márquez, Natalia Mena-Vázquez, Antonio Fernández-Nebro

**Affiliations:** 1 Instituto de Investigación Biomédica de Málaga y Plataforma en Nanomedicina-IBIMA Plataforma, BIONAND, Málaga, Spain; 2 UGC de Reumatología, Hospital Regional Universitario de Málaga, Málaga, Spain; 3 Departamento de Medicina, Universidad de Málaga, Málaga, Spain; 4 Unidad de Gestión Clínica de Endocrinología y Nutrición, Hospital Clínico Virgen de la Victoria, Málaga, Spain; 5 CIBER Fisiopatología de la Obesidad y Nutrición (CIBEROBN), Instituto de Salud Carlos III, Madrid, Spain; 6 Unidad de Bioinformática, Centro de Supercomputación y Bioinnovación (SCBI), Universidad de Málaga, Málaga, Spain; 7 Servicios Centrales de Apoyo a la Investigación (SCAI), Universidad de Málaga, Málaga, Spain

**Keywords:** anti-TNF, biomarker, inflammatory activity, miRNA, rheumatoid arthritis

## Abstract

**Introduction:**

Tumour necrosis factor inhibitors (anti-TNF agents) are a milestone in rheumatoid arthritis (RA) management, although 20-40% of RA patients do not achieve clinical remission. Identifying reliable biomarkers to predict the therapeutic response to anti-TNF agents remains an unmet clinical need.

**Methods:**

This study aimed to identify and validate plasma microRNAs (miRNAs) as biomarkers of response to anti-TNF treatment. A two-stage prospective observational (discovery and validation) study was performed. The study population comprised anti-TNF–naïve RA patients and sex- and age-matched healthy controls (HC). Whole miRNA expression was assessed using the Illumina NextSeq 550 platform, followed by bioinformatic analysis. Differentially expressed miRNAs identified in the discovery phase were quantified using real-time quantitative PCR in the validation cohort. Logistic regression analysis assessed associations between miRNA expression and response to anti-TNF treatment.

**Results:**

A total of 94 participants were included: 70 anti-TNF–naïve RA patients (discovery n = 28; validation n = 42) and 24 healthy controls (14 and 10, respectively). Clinical response at week 24 was defined as DAS28-CRP < 3.2 (responders) versus DAS28-CRP ≥ 3.2 (non-responders). Non-responders were characterised by higher inflammatory activity, poorer HAQ scores, and a higher prevalence of smoking. Forty-five miRNAs were differentially expressed between RA patients and HC, and 9 were expressed between responders and non-responders (*p* < 0.05). In the validation cohort, miR-134-5p differed between responders and non-responders (ΔCt_Responders_ = 4.088(±1.266); ΔCt_Non-Responders_ = 5.640(±2.872); ΔΔCt_RespondersVsNon-Responders_ = -1.552; Fold-Change = 2.932; *p* = 0.024). Logistic regression adjusted for age and sex showed higher miR-134-5p expression to be associated with lack of response to anti-TNF therapy (OR, 1.74; 95% CI: 1.02–2.93; *p* = 0.039).

**Conclusion:**

miR-134-5p may serve as a predictive biomarker of poor response to anti-TNF therapy in RA.

## Introduction

Rheumatoid arthritis (RA) is a chronic autoimmune disease characterised by persistent synovial inflammation, progressive joint damage, and functional disability (1). It has been estimated that RA affects 0.5-1% of the global population, with a marked female predominance (2). Despite extensive research, the molecular mechanisms underlying clinical heterogeneity, inflammatory activity, and response to treatment in RA patients remain unclear (3).

Current RA therapy comprises both synthetic and biologic disease modifying anti-rheumatic drugs (DMARDs). Tumour necrosis factor inhibitors (anti-TNF) in particular have advanced the management of RA by markedly improving prognosis and quality of life (4). However, 20-40% of RA patients treated with biological DMARDs do not achieve an adequate response owing to lack of efficacy, adverse events, or loss of response during treatment (5). Consequently, it is necessary to identify biomarkers that predict response to treatment and enable rheumatologists to select optimal therapy, avoid toxicities, and reduce healthcare costs.

MicroRNAs (miRNAs) are small (20–22 nucleotides long), highly conserved, endogenous non-coding RNA molecules. They post−transcriptionally regulate gene expression by binding to complementary sequences in the 3′−untranslated regions of target mRNAs (6). miRNAs are thought to regulate more than 60% of the protein-coding genes (7). Therefore, they play a key role in numerous biological processes such as immune activation, cell differentiation, and cytokine signalling. In recent years, miRNAs have emerged as promising diagnostic and prognostic biomarkers in autoimmune inflammatory diseases, including RA (8).

The literature shows that RA patients have altered miRNA expression profiles in blood, plasma, serum, and synovial fluid samples (9). Furthermore, specific miRNAs (miR-146a, miR-155, miR-223, and miR-125b) have been associated with severity of RA and response to DMARDs (10). However, the findings of most published articles are restricted by methodological limitations, small sample size, and marked heterogeneity between cohorts, thus complicating the validation of miRNA profiles as reproducible clinical biomarkers (8). Several studies have evaluated circulating miRNAs as predictors of anti-TNF effectiveness in RA, supporting their potential clinical utility but also highlighting important barriers to reproducibility. Reported signals and effect sizes have often varied across cohorts, likely due to differences in biospecimen type (serum vs plasma vs PBMC), pre-analytics (including haemolysis), profiling platforms (RT-qPCR vs NGS), normalization strategies, and response definitions at follow-up. More recent integrative approaches combining broader miRNome profiling with multivariable models and ROC/AUC-based evaluation suggest that predictive performance may improve when miRNAs are considered within clinical-molecular panels rather than as single markers (8, 11–13). These observations underscore the need for prospective studies with a discovery-validation structure and clearly defined endpoints to identify robust baseline circulating predictors of anti-TNF response.

Rheumatoid arthritis is clinically heterogeneous, and inter-individual differences in inflammatory pathways may contribute to variability in response to cytokine blockade. Diverse synovial inflammatory programs and incomplete regulation of NF-κB signalling illustrate mechanistic variability that could plausibly influence therapeutic response (14). Genomic studies also support a shared autoimmune architecture, implicating immune regulatory loci that may shape baseline immune set points and treatment outcomes (15). Heterogeneity is further evident in treatment response and toxicity to conventional therapy, including pharmacogenetic variability affecting methotrexate (16). Emerging immunothrombotic and immunometabolic mechanisms reinforce the need for blood-based predictors that integrate systemic biology (17, 18). In this context, circulating microRNAs represent attractive candidates, and recent computational approaches for biomarker discovery and panel optimization provide a methodological foundation for developing and evaluating circulating miRNA predictors of anti-TNF response (19–22).

Therefore, this study aimed to identify and validate baseline plasma miRNAs associated with clinical response to first anti-TNF therapy at week 24. As an exploratory analysis, we assessed within-patient changes from baseline to week 24 to evaluate potential on-treatment modulation.

## Patients and methods

### Study design

An observational prospective study was conducted. The study population comprised RA patients and sex- and age-matched healthy controls (HC). RA was diagnosed according to the 2010 criteria of the American College of Rheumatology/European League against Rheumatism (ACR/EULAR) classification criteria (23). Participants were consecutively recruited between June 2022 and June 2023 during routine visits to the Rheumatology Department of Hospital Universitario de Málaga, Málaga, Spain. Eligible patients were aged ≥ 16 years, naïve to biological therapy and had been prescribed their first anti-TNF treatment with moderate-to-high disease activity at baseline (28-joint Disease Activity Score [DAS28] ≥ 3.2), despite treatment with conventional synthetic disease-modifying antirheumatic drugs (csDMARDs). Patients with other rheumatic diseases, except secondary Sjögren’s syndrome, were excluded. The HC group comprised unrelated individuals from the same geographical area.

Written informed consent was obtained from all participants before enrolment. Both the study and the protocol fulfilled the tenets of the Declaration of Helsinki. The study was approved by the Research Ethics Committee of Málaga (“Comité de Ética de la Investigación de Málaga”), with identification code: PI22/01207.

All participants underwent clinical and analytical evaluations at two time-points: prior to initiation of anti-TNF treatment (baseline visit, W0) and after 24 weeks of treatment (follow-up, W24). At both time-points, peripheral blood samples were obtained from individuals and were collected in K2-EDTA tubes (#12977696, Beckton Dickson, NJ, USA). Rapidly, samples were centrifuged at 2000*g* for 15 min, the plasma was aliquoted in cryotubes (#C3236-50EA, Nunc, Thermo Fisher Scientific, NY, USA) and finally stored at −80 °C until their use.

Following published recommendations for RNA-seq studies involving human samples (24, 25), the discovery cohort comprised 14 individuals per group at each time-point: responders (n = 14), non-responders (n = 14), and HC (n = 14). An independent validation cohort comprised 52 individuals: 42 RA patients and 10 HC.

### Sociodemographic and clinical variables

Demographic, clinical, analytical, and treatment-related data were collected by rheumatologists during routine clinic practice. All data were registered in a secure database following a standardized protocol.

Demographic variables included age (years), sex, and ethnic background. Cardiovascular risk variables were recorded. These included smoking (smoker or non-smoker), obesity (body mass index ≥ 30 kg/m²), high blood pressure (defined as ≥ 140/90 mmHg or ongoing antihypertensive treatment), diabetes mellitus diagnosed according to American Diabetes Association criteria (26), dyslipidaemia (total cholesterol > 200 mg/dL, LDL cholesterol > 160 mg/dL, or triglycerides > 175 mg/dL), and history of cardiovascular disease.

For RA patients, the date of symptom onset, date of diagnosis, and disease duration were recorded, as were a series of laboratory parameters: rheumatoid factor (RF) (reference value [RV] 20 U/mL; high titres > 60 U/mL), anti–cyclic citrullinated peptide antibodies (ACPA) (RV 10 U/mL; high titres > 340 U/mL), C-reactive protein (CRP, mg/dL), and erythrocyte sedimentation rate (ESR, mm/h).

The cumulative inflammatory burden from diagnosis to W0 was estimated as the mean of all DAS28 values recorded over of the disease course. After 24 weeks of anti-TNF treatment, disease activity was categorized/defined as low-remission (DAS28-CRP < 3.2) or moderate-high (DAS28-CRP ≥ 3.2). Disease severity variables were also documented, including the presence of radiographic erosions and physical disability, assessed based on the mean score on the Spanish version of the Health Assessment Questionnaire (HAQ) (27) during the course of the disease.

Concomitant treatment with csDMARDs (methotrexate, leflunomide, sulfasalazine, or hydroxychloroquine) and glucocorticoids, including the dose administered at each visit, was also recorded.

### miRNA expression

Total plasma miRNA was isolated using the RNeasy Serum/Plasma Advanced Kit (#217204, Qiagen, Hilden, Germany) following the manufacturer’s instructions. 10 pM of cel-miR-39-3p spike-in or exogenous control (Assay 478293_mir, #10620310, Thermo Fisher Scientific, Waltham, MA, USA) was added to each sample to monitor extraction efficiency. Then, purity and quality were evaluated using the NanoPhotometer^™^ spectrophotometer (IMPLEN, CA, USA), the concentration was determined using the Qubit RNA Assay Kit (#Q32852, Invitrogen, MA, USA) with the Qubit 3.0 fluorometer (Invitrogen), and the integrity of the concentration was assessed using the RNA Nano 6000 Assay Kit (#5067-1511, Agilent Technologies, CA, USA) with the Agilent 2100 Bionalyzer system (Agilent Technologies).

For the discovery phase, prior to miRNA-seq, 1 µg of total RNA sample was spent to prepare small RNA libraries using the NEBNext^®^ Multiplex Small RNA Library Prep Set for Illumina^®^ (#E7560S, New England Biolabs, MA, USA) following the manufacturer’s protocol. Libraries were then pooled and sequenced on the Illumina NextSeq 550 platform (Illumina, CA, USA), generating 75-bp single-end (SE) reads with a minimum depth of 5–10 million reads per sample.

Once miRNA-seq results were analysed, differential miRNA expression profiles between groups of individuals were identified. In order to select the miRNAs for the validation study, the combined criteria of statistical significance, magnitude of the change in expression, and biological relevance were applied (11). First, miRNAs were ranked based on the adjusted significance value obtained in comparisons between the different groups. Next, a fold change threshold was established: ≥ 2 for over-expressed miRNAs and ≤ 0.5 for under-expressed miRNAs. Finally, miRNAs whose target genes were involved in biological functions related to the pathophysiology of RA and response to treatment, according to the available literature, were prioritised. The selected housekeeping miRNAs were hsa-miR-3184-5p, hsa-miR-128-3p, and hsa-let-7d-3p.

The expression of the miRNAs identified as candidates in the discovery phase was assessed using quantitative polymerase chain reaction (PCR) with specific probes for each miRNA. Reverse transcription of miRNA was performed with a TaqMan Advanced miRNA cDNA Synthesis Kit (#A28007, Thermo Fisher Scientific) using 2 µL of isolated miRNA, according to the manufacturer’s protocol, on a C1000 Touch PCR thermal cycler (BioRad, Hamburg, Germany). The relative expression of miRNA in serum/plasma was determined with TaqMan microRNA assays (**Supplementary Table 1**). RT-qPCR was performed on a Quant Studio 12K instrument (Thermo Fisher Scientific) using TaqMan Fast Advanced Master Mix (#4444557, Thermo Fisher Scientific) following the manufacturer’s protocol. The reaction was performed in triplicate under the following conditions: 95 °C for 20 s, 40 × 1 s at 95 °C, and 20 s at 60 °C. The same RT-qPCR protocol was followed for the exogenous control and markers of haemolysis. The relative expression of each candidate miRNA was calculated by delta delta Ct method (28), following MIQE guidelines (29). Briefly, the mean Ct value of the 3 endogenous miRNAs (miR-3184-5p, miR-128-3p, and let-7d-3p) was calculated to obtain the delta Ct of each candidate miRNA (ΔCt = Ct endogenous – Ct target). Then, the delta delta Ct was assessed as ΔΔCt = ΔCt group A – ΔCt group B. Finally, the fold change was calculated (Fold-change = 2^-ΔΔCt^).

### Markers of haemolysis

Because haemolysis can release erythrocyte−derived miRNAs that markedly alter circulating miRNA profiles in both serum and plasma samples, its presence was systematically evaluated. Among the different methods for assessing haemolysis, the relationship between hsa-miR-451a and hsa-miR-23a-3p is the most sensitive for detecting haemolysis levels as low as 0.001% in serum. Therefore, this approach was selected and performed as previously described (30). Haemolysis was assessed using the ΔCq approach based on miR-23a-3p and miR-451a (ΔCq = Cq(miR-23a-3p) − Cq(miR-451a)), applying published thresholds (<5 low, 5–7 moderate, >7 high). Samples exceeding ΔCq > 7 were prespecified for exclusion, and ΔCq distributions were compared across study groups.

### Bioinformatic analysis

A standardized bioinformatic pipeline was employed for the analysis of miRNA-seq results. Quality control of raw single-end reads was performed using FastQC (v0.12.0). Adapter trimming and removal of low-quality reads were carried out with fastp (v1.0.1.16), and post-filtering FastQC reports were inspected to confirm the expected small RNA size distribution. Cleaned reads were aligned using Bowtie2 (v2.0.2) in local alignment mode with the very sensitive local preset. Alignments were performed against the human miRNA reference from miRBase (release v22; 1,917 annotated miRNAs), and in parallel against a microbial small RNA database as described above. Bowtie2 default reporting (best alignment per read) was used. Read counts per miRNA were summarized from SAM/BAM files using the Sam2count.py script to generate the miRNA-by-sample count matrix; alignment summaries (uniquely mapped, multi-mapped, and unaligned reads) were retained and reported for transparency. Sequencing depth and alignment performance were assessed across samples (read depth per sample, percentage mapped to miRNAs, and proportions of uniquely mapped, multi-mapped, and unaligned reads). To evaluate potential technical effects, principal component analysis (PCA) of normalized counts was performed; no clustering consistent with batch effects was observed. Differential miRNA expression between groups was analysed using DESeq2 with a negative binomial model and Benjamini–Hochberg false discovery rate (FDR) correction. Differentially expressed miRNAs (miDE) were defined as those with adjusted p < 0.05 and |fold change| ≥ 2.

### Statistical analysis

A descriptive analysis was performed for all demographic and clinical variables. Categorical variables were expressed as absolute frequencies and percentages, and continuous variables as means (standard deviation, SD) or medians (interquartile range, IQR), as appropriate. Normality was assessed using the Kolmogorov-Smirnov test.

Comparisons of clinical, laboratory, and miRNA variables between RA patients and HC, as well as between responders and non-responders, were performed using Pearson’s χ² test or the *t* test, as appropriate. Discriminative performance of the models was assessed using receiver operating characteristic (ROC) curves, and the area under the curve (AUC) with 95% confidence intervals (CI) was reported.

Logistic regression analyses were conducted to identify miRNAs independently associated with a diagnosis of RA and with response to treatment among RA patients. All statistical analyses were performed using IBM SPSS Statistics for Mac OS, Version 28 (IBM Corp., Armonk, NY, USA).

## Results

### Cohort characteristics

**Figure 1:**
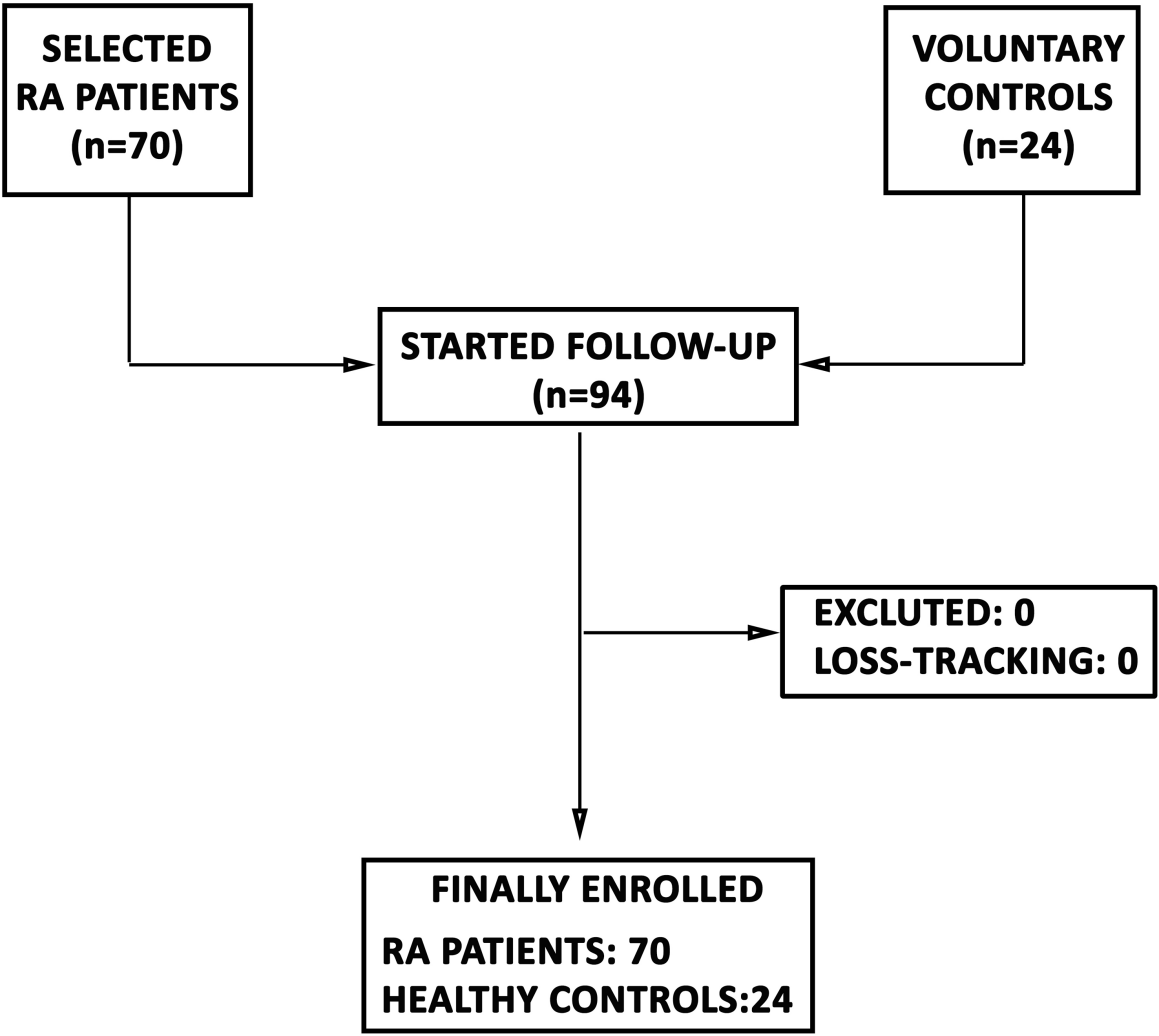
STROBE flow-chart of patients’ recruitment.

The study population comprised 94 participants: 70 RA patients and 24 HCs (**Figure 1**). All individuals were allocated to two independent cohorts: a discovery cohort (28 RA, 14 HC) and a validation cohort (42 RA, 10 HC). **Table 1** summarizes the epidemiological and clinical characteristics of both cohorts.

**Table 1:** Epidemiological and clinical characteristics of the discovery and validation cohorts.

Variable	Discovery cohort			Validation cohort		
	RA (n = 28)	HC (n = 14)	*p*-value	RA (n = 42)	HC (n = 10)	*p*-value
Epidemiological						
Female sex, n (%)	24 (85.7)	12 (85.7)	1.000	33 (78.6)	8 (80.0)	0.921
Age (in years), mean (SD)*	57.1 10.4)	57.3 (5.5)	0.944	55.6 (13.4)	56.2 (11.1)	0.893
Caucasian, n (%)	28 (100.0)	14 (100.0)	1.000	42 (100.0)	10 (100.0)	1.000
Educational level			0.657			0.993
Basic education, n (%)	7 (25.0)	5 (35.7)		12 (28.6)	3 (30.0)	
Non-university higher education, n (%)	14 (50.0)	5 (35.7)		21 (50.0)	5 (50.0)	
University education, n (%)	7 (25.0)	4 (28.6)		9 (21.4)	2 (20.0)	
BMI, kg/m^2^, mean (SD)	28.0 (4.9)	28.9 (3.9)	0.572	26.9 (3.7)	26.2 (5.6)	0.664
Comorbidities						
Dyslipidaemia, n (%)	6 (21.4)	0 (0.0)	0.061	10 (23.8)	1 (10.0)	0.337
Hypertension, n (%)	7 (25.0)	2 (14.3)	0.425	11 (26.2)	4 (40.0)	0.386
Smoking habit			0.081			0.048
Non-smoking, n (%)	20 (71.4)	12 (85.7)		32 (76.2)	9 (90.0)	
Smoking, n (%)	8 (28.6)	2 (14.3)		10 (23.8)	1 (10.0)	
Diabetes mellitus, n (%)	4 (14.3)	2 (14.3)	1.000	4 (9.5)	0 (0.0)	0.310
Obesity, n (%)	13 (46.4)	4 (28.6)	0.266	7 (16.7)	2 (20.0)	0.802
Clinical						
Disease duration, months, median (IQR)	54.1 (126.6)	–	–	85.8 (161.5)	–	–
Diagnostic delay, months, median (IQR)	7.4 (9.0)	–	–	5.4 (6.8)	–	–
Erosions, n (%)	14 (50.0)	0 (0.0)	0.001	20 (47.6)	0 (0.0)	0.005
RF+ (> 10 U/mL), n (%)	26 (92.9)	0 (0.0)	<0.001	34 (81.0)	0 (0.0)	<0.001
ACPA+ (> 20 U/mL), n (%)	23 (82.1)	0 (0.0)	<0.001	33 (78.6)	0 (0.0)	<0.001
ACPA > 340 U/mL, n (%)	10 (35.7)	0 (0.0)	0.010	10 (23.8)	0 (0.0)	0.086
DAS28-CRP at baseline, mean (SD)	5.1 (0.9)	–	–	4.7 (0.9)	–	–
DAS28-CRP, average, mean (SD)	3.7 (0.9)	–	–	3.5 (0.8)	–	–
HAQ, median (IQR)	1.5 (0.7)	–	–	1.2 (0.7)	–	–
HAQ, average, mean (SD)	1.1 (0.5)	–	–	1.1 (0.6)	–	–
Treatment						
Methotrexate, n (%)	20 (71.4)	0 (0.0)	<0.001	25 (59.5)	0 (0.0)	<0.001
Hydroxychloroquine, n (%)	4 (14.3)	0 (0.0)	0.137	7 (16.7)	0 (0.0)	0.165
Leflunomide, n (%)	4 (14.3)	0 (0.0)	0.137	7 (16.7)	0 (0.0)	0.165
Sulfasalazine, n (%)	3 (10.7)	0 (0.0)	0.204	16 (38.1)	0 (0.0)	0.019
Glucocorticoids, n (%)	21 (75.0)	0 (0.0)	<0.001	31 (73.8)	0 (0.0)	<0.001
Glucocorticoids, median (IQR)	7.5 (3.8)	–	–	5.0 (2.5)	–	–

RA, rheumatoid arthritis; HC, healthy controls; SD, standard deviation; BMI, body mass index; IQR, interquartile range; RF, rheumatoid factor; ACPA, anti–citrullinated peptide antibody; DAS28, 28-joint Disease Activity Score; CRP, C-reactive protein; HAQ, Health Assessment Questionnaire.

No significant differences were found between RA patients and HC in either cohort regarding demographic variables, including sex (*p* = 0.921), age (*p* = 0.893), ethnicity (*p* = 1.000), educational level (*p* = 0.993), and body mass index (*p* = 0.664). Among clinical and comorbidity variables, only smoking status differed significantly, being more frequent in RA patients (*p* = 0.048).

As expected, RA patients had positive RF and ACPA antibody titres and radiographic erosions, consistent with established disease. Regarding pharmacological treatment, all RA patients received csDMARDs and glucocorticoids, whereas these medications were absent in HC.

All samples included in circulating miRNA analyses were within the low-haemolysis range based on ΔCq (miR-23a-3p − miR-451a), and no samples met the prespecified exclusion criterion (ΔCq > 7). ΔCq values did not differ between groups (ANOVA p = 0.608), indicating no evidence of differential haemolysis.

After 24 weeks of anti-TNF therapy, RA patients were classified as responders (DAS28-CRP < 3.2) or non-responders (DAS28-CRP ≥ 3.2). As expected, non-responders had higher cumulative mean (SD) DAS28-CRP scores than responders in both cohorts (discovery cohort, 4.1 [1.0] vs 3.3 [0.5], *p* = 0.024; validation cohort, 4.1 [0.6] vs 3.2 [0.7], *p* = 0.004), consistent with more persistent disease activity (**Table 2**).

**Table 2:** Epidemiological and clinical characteristics of responders and non-responders.

Variable	Discovery cohort			Validation cohort		
	Responders (n = 14)	Non-responders (n = 14)	*p*-value	Responders (n = 29)	Non-responders (n = 13)	*p*-value
Epidemiological						
Female sex, n (%)	12 (85.7)	12 (85.7)	1.000	24 (82.8)	9 (69.2)	0.323
Age (in years), mean (SD)	56.1 (10.6)	58.1 (10.5)		55.6 (12.3)	55.6 (16.0)	0.996
Caucasian, n (%)	14 (100.0)	14 (100.0)	1.000	29 (100.0)	13 (100.0)	1.000
Educational level			0.867			0.600
Basic education, n (%)	4 (28.6)	3 (21.4)		16 (55.2)	3 (23.1)	
Non-university higher education, n (%)	7 (50.0)	7 (50.0)		5 (17.2)	8 (61.5)	
University education, n (%)	3 (21.4)	4 (28.6)		8 (27.6)	2 (15.4)	
BMI, kg/m^2^, mean (SD)	29.1 (4.8)	27.0 (4.9)		27.0 (4.0)	26.5 (3.2)	0.683
Comorbidities						
Dyslipidaemia, n (%)	3 (21.4)	3 (21.4)	1.000	6 (20.7)	4 (30.8)	0.478
Hypertension, n (%)	4 (28.6)	3 (21.4)	0.663	8 (27.6)	3 (23.1)	0.759
Smoking habit			0.352			0.016
Non-smoking, n (%)	11 (78.6)	9 (64.3)		16 (55.2)	3 (23.1)	
Smoking, n (%)	3 (21.4)	5 (35.7)		13 (44.8)	10 (76.9)	
Diabetes mellitus, n (%)	2 (14.3)	2 (14.3)	1.000	3 (10.3)	1 (7.7)	0.787
Obesity, n (%)	7 (50.0)	5 (35.7)	0.659	5 (17.2)	2 (15.4)	0.881
Clinical						
Disease duration, months, median (IQR)	59.3 (100.4)	53.2 (173.5)	0.734	83.2 (138.5)	83.6 (215.7)	0.155
Diagnostic delay, months, median (IQR)	6.8 (8.7)	10.1 (11.5)	0.125	5.7 (5.9)	7.0 (11.4)	0.629
Erosions, n (%)	7 (50.0)	7 (50.0)	1.000	12 (41.4)	8 (61.5)	0.227
RF+ (> 10 U/mL), n (%)	13 (92.9)	13 (92.9)	1.000	23 (79.3)	11 (84.6)	0.686
ACPA+ (> 20 U/mL), n (%)	14 (100.0)	9 (64.3)	0.014	21 (84.0)	9 (75.9)	0.513
ACPA > 340 U/mL, n (%)	5 (35.7)	5 (35.7)	1.000	10 (34.5)	0 (0.0)	0.015
DAS28-CRP at baseline, mean (SD)	5.2 (0.7)	5.4 (1.1)	0.744	4.6 (0.9)	4.9 (1.1)	0.376
DAS28-CRP, average, mean (SD)	3.3 (0.5)	4.1 (1.0)	0.024	3.2 (0.7)	4.1 (0.6)	0.004
HAQ, median (IQR)	1.5 (0.7)	1.3 (0.9)	0.571	1.2 (0.7)	1.5 (0.7)	0.232
HAQ, average, mean (SD)	1.0 (0.6)	1.2 (0.5)	0.487	0.9 (0.6)	1.4 (0.4)	0.031
Treatment						
Methotrexate, n (%)	10 (71.4)	10 (71.4)	1.000	15 (51.7)	10 (76.9)	0.124
Hydroxychloroquine, n (%)	3 (21.4)	1 (7.1)	0.280	6 (20.7)	1 (7.7)	0.296
Leflunomide, n (%)	1 (7.1)	3 (21.4)	0.280	4 (13.8)	3 (23.1)	0.455
Sulfasalazine, n (%)	0 (0.0)	3 (21.4)	0.067	14 (48.3)	2 (15.4)	0.042
Glucocorticoids, n (%)	10 (71.4)	11 (78.6)	0.663	21 (72.4)	10 (76.9)	0.759
Glucocorticoids, median (IQR)	6.2 (2.5)	7.5 (5.0)	0.605	5.0 (2.5)	5.0 (1.3)	0.983

RA, rheumatoid arthritis; SD, standard deviation; BMI, body mass index; IQR, interquartile range; RF, rheumatoid factor; ACPA, anti–citrullinated peptide antibody; DAS28, 28-joint Disease Activity Score; CRP, C-reactive protein; HAQ, Health Assessment Questionnaire.

In the validation cohort, non-responders also had higher mean (SD) HAQ scores than responders (1.4 [0.4] vs 0.9 [0.6], *p* = 0.031), reflecting poorer physical function. In the discovery cohort, the proportion of ACPA-positive patients was significantly lower among non-responders than responders (64.3% vs 100.0%, *p* = 0.014). Additionally, in the validation cohort, smoking prevalence was significantly higher among non-responders than responders (76.9% vs 44.8%, *p* = 0.016).

### miRNA-seq analysis

Across all small RNA-seq libraries, total read depth ranged from ~1M to >20M reads per sample, with miRNA mapping rates between ~1.9% and ~22.3%. The distribution of uniquely mapped, multi-mapped, and unaligned reads per sample is summarized in **Supplementary Table 2**, and no systematic differences in mapping efficiency were observed between experimental groups. Principal component analysis (PCA) of normalized miRNA counts (PC1 = 12% and PC2 = 9% variance explained) did not show clustering consistent with technical effects (**Supplementary Figure 1**), supporting that downstream differential expression results are unlikely to be driven by upstream processing variability.

In the discovery cohort, differential expression analysis identified 45 miRNAs that were significantly deregulated between RA patients and HC (**Table 3**), suggesting their potential role as diagnostic biomarkers in RA. The differentially expressed miRNAs include miRNAs previously related to oxidative stress, rheumatic diseases such as psoriatic arthritis, conditions secondary to RA such as atherosclerosis and cardiovascular risk, inflammatory processes, and B-cell autoreactivity.

**Table 3:** Differently expressed miRNAs in RA patients compared to HC.

miRNA	Expression	Log fold-change	*p*-value (adjusted)
hsa-miR-379-5p	Elevated	2.187	0.0051
hsa-miR-3184-3p	Elevated	1.200	0.0007
hsa-miR-409-3p	Elevated	1.898	0.0025
hsa-miR-657	Elevated	2.622	0.0136
hsa-miR-493-3p	Elevated	1.785	0.0421
hsa-miR-574-5p	Elevated	1.238	0.0103
hsa-miR-423-5p	Elevated	1.177	0.0008
hsa-miR-378c	Elevated	3.970	0.0008
hsa-miR-320c	Elevated	2.008	0.0005
hsa-miR-7704	Elevated	4.067	0.0288
hsa-miR-134-5p	Elevated	2.245	0.0073
hsa-miR-370-3p	Elevated	2.457	1.71823E-06
hsa-miR-378a-3p	Elevated	1.127	0.0183
hsa-miR-485-5p	Elevated	2.147	0.0117
hsa-miR-4446-3p	Elevated	1.618	0.0137
hsa-miR-433-3p	Elevated	3.593	0.0133
hsa-miR-320a-3p	Elevated	1.220	0.0421
hsa-miR-1307-3p	Elevated	1.733	0.0002
hsa-miR-1908-5p	Elevated	1.737	0.0179
hsa-miR-320b	Elevated	1.990	0.0007
hsa-miR-193b-5p	Elevated	1.758	0.0285
hsa-miR-744-5p	Elevated	1.037	0.0017
hsa-miR-320d	Elevated	1.996	0.0084
hsa-miR-3168	Elevated	4.213	0.0002
hsa-miR-127-3p	Elevated	1.514	0.0109
hsa-miR-193a-5p	Elevated	2.529	1.71823E-06
hsa-miR-142-3p	Elevated	2.603	0.0073
hsa-miR-483-5p	Elevated	4.404	4.0191E-09
hsa-miR-30a-5p	Reduced	-1.760	0.0066
hsa-miR-30e-5p	Reduced	-1.435	0.0051
hsa-miR-26b-5p	Reduced	-1.103	0.0022
hsa-miR-328-3p	Reduced	-1.254	0.0170
hsa-miR-182-5p	Reduced	-1.999	0.0147
hsa-miR-144-3p	Reduced	-2.383	0.0328
hsa-miR-486-3p	Reduced	-1.166	0.0103
hsa-miR-92a-3p	Reduced	-1.148	0.0007
hsa-miR-186-5p	Reduced	-1.536	0.0009
hsa-miR-16-5p	Reduced	-2.103	0.0022
hsa-let-7b-3p	Reduced	-3.145	0.0170
hsa-miR-363-3p	Reduced	-1.852	7.5162E-06
hsa-miR-3173-5p	Reduced	-4.170	0.0325
hsa-miR-20a-5p	Reduced	-3.412	0.0117
hsa-miR-1275	Reduced	-4.002	0.0334
hsa-miR-223-3p	Reduced	-2.861	0.0020
hsa-miR-486-5p	Reduced	-1.094	0.0288

RA, rheumatoid arthritis; miRNA, micro-RNA.

Subsequent analyses comparing responder RA patients at W0 and W24 revealed no significant intra-individual differential expression in any miRNA (data not shown).

Finally, the comparative analysis between responders and non-responders at W0 revealed 9 miRNAs exhibiting significant differences in miRNA expression between the groups (**Table 4**). These miRNAs represent potential biomarkers associated with response to anti-TNF treatment. Furthermore, the target genes for these miRNAs were *TP73*, *MAPK3*, *MYC*, and *DLX5*. Notably, miR-134-5p was not differentially expressed between responders and non-responders in the discovery miRNA-seq analysis (**Table 4**); its association with treatment response was observed in the RT-qPCR validation cohort.

**Table 4:** Differentially expressed miRNAs in responders and non-responders.

miRNA	Expression	Log fold-change	*p*-value (adjusted)	Gene (target)
hsa-miR-3184-3p	Reduced	-1.1211	0.0364	Unknown
hsa-miR-423-5p	Reduced	-1.0949	0.0405	Unknown
hsa-miR-378c	Reduced	-4.7019	0.0015	Unknown
hsa-miR-370-3p	Reduced	-1.8704	0.0184	Unknown
hsa-miR-1307-3p	Reduced	-1.4778	0.0405	Unknown
hsa-miR-320b	Reduced	-1.9942	0.0184	*DLX5*; *MYC*
hsa-miR-3168	Reduced	-5.1928	0.0001	Unknown
hsa-miR-193a-5p	Reduced	-2.4260	0.0004	*TP73*
hsa-miR-483-5p	Reduced	-3.8457	0.0001	*MAPK3*

RA, rheumatoid arthritis; miRNA, micro-RNA.

### Validation of miRNA-seq results by RT-qPCR

In the discovery phase, miRNA-seq analysis revealed differences in expression profiles between RA patients and HC, as well as between responders and non-responders. Seven miRNAs were selected for validation based on combined selection criteria⁠ (including statistical significance, fold−change ≥ 2 or ≤ 0.5, and biological relevance to pathways implicated in the pathophysiology of RA and the response to anti−TNF therapy⁠).

Validation by RT-qPCR showed no significant differences in expression levels of any miRNA between RA patients and HC (**Table 5**). Meanwhile, among the selected candidates, expression of miR-134-5p significantly differ in non-responders compared with responders (ΔCt_Responders_ = 4.088(± 1.266); ΔCt_Non-Responders_ = 5.640(± 2.872); ΔΔCt_RespondersVsNon-Responders_ = -1.552; Fold-Change = 2.932; *p* = 0.0246). ROC analysis of miR-134-5p showed modest discrimination (AUC = 0.611, 95% CI 0.47–0.74; p = 0.120).

**Table 5:** Results of the analysis for validation of miRNA expression by RT-qPCR.

miRNA	RA patients (n = 42)	HC (n = 10)	*p*-value	Responders (n = 29)	Non-responders (n = 13)	*p*-value
miR-106b-5p	-0.371 (± 1.468)	-0.234 (± 1.128)	0.785	-0.542 (± 1.576)	-0.068 (± 1.243)	0.339
miR-92a-3p	-4.905 (± 1.284)	-4.957 (± 0.882)	0.905	-5.057 (± 1.424)	-4.634 (± 0.959)	0.328
miR-223-3p	-5.605 (± 1.220)	-5.266 (± 0.833)	0.412	-5.719 (± 1.332)	-5.403 (± 0.991)	0.444
miR-451-a	-5.828 (± 1.904)	-5.966 (± 1.056)	0.827	-5.924 (± 2.076)	-5.657 (± 1.593)	0.679
miR-16-5p	-3.009 (± 1.512)	-3.259 (± 0.781)	0.617	-3.139 (± 1.676)	-2.776 (± 1.170)	0.478
miR-142-3p	0.028 (± 1.635)	0.542 (± 1.636)	0.379	-0.104 (± 1.529)	0.263 (± 1.854)	0.509
miR-134-5p	4.567 (± 2.080)	4.792 (± 1.899)	0.853	4.088 (± 1.266)	5.640 (± 2.872)	**0.025**

*t* test results are presented as ΔCt [mean (± SD)] for each miRNA. RA, rheumatoid arthritis; HC, healthy controls; miRNA, micro-RNA.

A logistic regression model adjusted for age and sex established a significant association between higher miR-134-5p expression levels and the lack of therapeutic response to anti-TNF treatment (OR [95%CI], 1.74 [1.02-2.93]; *p* = 0.039) (**Table 6**).

**Table 6:** Results of the study of the association of miRNAs with RA and therapeutic response in a logistic regression model.

miRNA	RA patients vs HC	Responders vs non-responders
miR-106b-5p	R^2^ = 0.010 OR: 0.965, 95%CI (0.59-1.57) *p* = 0.887	R^2^ = 0.046 OR: 1.166, 95%CI (0.72-1.87) *p* = 0.527
miR-92a-3p	R^2^ = 0.018 OR: 1.180, 95%CI (0.62-2.20) *p* = 0.609	R^2^ = 0.043 OR: 1.184, 95%CI (0.65-2.14) *p* = 0.576
miR-223-3p	R^2^ = 0.015 OR: 0.895, 95%CI (0.52-1.51) *p* = 0.681	R^2^ = 0.034 OR: 1.058, 95%CI (0.64-1.73) *p* = 0.825
miR-451-a	R^2^ = 0.017 OR: 1.099, 95%CI (0.73-1.63) *p* = 0.641	R^2^ = 0.033 OR: 0.978, 95%CI (0.66-1.43) *p* = 0.910
miR-16-5p	R^2^ = 0.021 OR: 1.209, 95%CI (0.74-1.97) *p* = 0.446	R^2^ = 0.032 OR: 1.018, 95%CI (0.64-1.60) *p* = 0.939
miR-142-3p	R^2^ = 0.012 OR: 0.895, 95%CI (0.63-1.25) *p* = 0.522	R^2^ = 0.043 OR: 1.096, 95%CI (0.79-1.50) *p* = 0.570
miR-134-5p	R^2^ = 0.011 OR: 0.885, 95%CI (0.58-1.34) *p* = 0.565	R^2^ = 0.208 OR: 1.738, 95%CI (1.02-2.93) *p* = 0.039*

RA, rheumatoid arthritis; HC, healthy controls; miRNA, micro-RNA. *p < 0.05 after including age and sex in the logistic regression model.

Finally, to address potential confounding due to baseline clinical differences, we repeated the logistic regression analyses using expanded adjustment sets. Fully adjusted models for all candidate miRNAs are shown in **Supplementary Table 3**. In these models, miR-134-5p remained independently associated with non-response after adjustment for age, sex, smoking status, baseline DAS28-CRP, and baseline glucocorticoid use (adjusted OR 1.641; 95% CI 1.002–2.677; p = 0.043). A dedicated model including baseline clinical covariates alongside miR-134-5p is provided in **Supplementary Table 4** (Nagelkerke R² = 0.256).

## Discussion

Anti-TNF treatment has significantly advanced management of RA, improving affected patients’ quality of life and physical condition. However, a high percentage of patients do not achieve clinical remission after anti-TNF treatment. Therefore, it is clear that specific and sensitive biomarkers are needed to predict therapeutic response and optimize treatment selection (31). In this context, miRNAs have been proposed as promising biomarkers owing to their stability in biological fluids and the role played in both immunological and inflammatory processes in RA. Therefore, we present an observational and exploratory study aiming to identify circulating plasma miRNAs as potential novel biomarkers in RA.

miRNA-seq was carried out on a discovery cohort in order to identify miRNA signatures when comparing different groups: RA patients vs HCs, and responder vs non-responder RA patients. A 45-miRNA signature was identified as differentially expressed between RA patients and HCs. Previous literature establishes that several of these miRNAs are involved in mechanistic processes and pathways that are important for the development of RA. Specifically, miR-423-5p and miR-320a-3p have been associated with apoptosis, oxidative stress, and cardiovascular disease (32, 33), miR-370-3p with oxidative stress (34), miR-30a-5p with promotion of B-cell hyperactivity (35), and miR-142-3p and miR-223-3p with both inflammation and RA (36–38). Furthermore, after comparing responders and non-responders at baseline a miRNA signature with significant differential expression was identified. This points to a putative baseline molecular fingerprint associated with subsequent anti−TNF response. The currently reported target genes for these miRNAs are *TP73*, *MAPK3*, *MYC*, and *DLX5*, which are implicated in pathways central to rheumatoid synovitis and anti-TNF–modulated inflammation. *TP73* is relevant in DNA-damage response and apoptosis (*TP73*) (39, 40), and the activation of p73, protein encoded by *TP73* gene, has been proposed as a therapeutic strategy in RA (41). *MAPK3*, also known as *ERK1*, plays a central role in the MAPK/ERK pathway signalling which is related to RA pathogenesis and inflammatory response (42). *MYC* plays an important role in B-cell homeostasis maintenance as a component of the B-cell receptor (43), this homeostasis is dysregulated in RA. *DLX5* has been recently related to inflammation-induced pain in RA patients (44).The longitudinal analysis of responders (pre− and post−therapy) did not reveal significant changes in miRNA expression, suggesting that these circulating miRNAs are not acutely modulated by anti−TNF therapy over 24 weeks but may instead reflect upstream regulatory states that could predispose to therapeutic non−response.

In the independent RT-qPCR validation cohort, 7 candidate miRNAs were selected (miR-106b-5p, miR-92a-3p, miR-223-3p, miR-451-a, miR-16-5p, miR-142-3p, and miR-134-5p) based on combined statistical and biological criteria derived from discovery-phase profiling, ensuring both effect size and mechanistic plausibility. No candidate reached statistical significance in the comparison of RA patients and HCs, whereas miR-134-5p was the only miRNA to be differentially expressed between clinical responders (ΔCt_Responders_ = 4.088(± 1.266); ΔCt_Non-Responders_ = 5.640(± 2.872); ΔΔCt_RespondersVsNon-Responders_ = -1.552; Fold-Change = 2.932; *p* = 0.0246). Consistently, a logistic regression model adjusted for sex and age showed that higher miR-134-5p expression was associated with lack of clinical response to anti-TNF therapy (OR [95%CI], 1.74 [1.02-2.93]; *p* = 0.039). Notably, non-responders also had higher inflammatory activity, worse functional status (HAQ), and greater smoking prevalence, indicating a more adverse phenotype. This pattern underscores the clinical relevance of identifying molecular predictors that can complement conventional risk indicators during selection of biologic therapy.

Although miR−134−5p has not been directly linked to RA aetiology in human studies to date, several lines of evidence have linked this miRNA to central processes in inflammatory, autoimmune regulation, and cell-types impaired in RA (45). It has been communicated that miR-134-5p target the KRAS signalling pathway, affecting T-cell activation threshold and enabling responses to autoantigens (46). Evidence supports that miR-134-5p can interface with innate immune signalling as has appeared within exploratory circulating−miRNA panels in autoimmune diseases, such as Sjögren’s syndrome or Still’s disease. It has been reported the involvement of miR-134-5p in both Sjögren’s syndrome with neuropathy and rheumatic heart disease, pointing to broader immunoinflammatory involvement (47, 48). Furthermore, Liao et al. associated elevated circulating miR−134 (isoform not specified) with disease activity in adult−onset Still’s disease, together with activation of TLR3 and downregulation of IL−18BP (49), an axis that could raise free IL−18 and perpetuate systemic inflammation as it is widely known to occur in RA (50). Beyond inflammation and autoimmunity, studies in animal models addressing nociceptive pathways, suggest a potential relevance of miR-134-5p in pain phenotypes (51, 52), commonly accompanying RA. Atherosclerosis leading to cardiovascular disease is the main cause of mortality and morbidity in RA patients (53). In atherosclerosis−related cellular models, miR−134 promotes lipid accumulation and macrophage inflammatory responses by inhibiting *ANGPTL4*, thereby augmenting lipoprotein lipase activity and pro−inflammatory cytokine secretion (54).

Taken together, these data support biological plausibility for miR−134−5p as a regulator at the interface of innate sensing, cytokine amplification, and myeloid activation, pathways that are associated with response to anti−TNF therapy. Moreover, our results support that circulating miRNA signatures can stratify therapeutic efficacy in RA and other conditions (8, 11–13).

This study presents several strengths, including its prospective design, two-stage strategy (discovery and validation), the use of standardized biological samples, and the detailed clinical evaluation of the individuals included. Nevertheless, several limitations warrant consideration. First, the sample size may limit the power to detect modest effect sizes and to validate additional miRNA candidates beyond miR-134-5p. To mitigate this, the discovery thresholds combined effect size and biological plausibility, and an independent cohort was used for validation by RT-qPCR. It was possible to identify differences between groups in both cohorts that are in line with the literature, to analyse several miRNAs related to RA, and to validate miR-134-5p as a novel potential biomarker for response to anti-TNF treatment. Still, multicentre replication with larger samples is required to refine estimates and assess robustness across care settings. Furthermore, miR-134-5p was validated in a modest sample; thus, this result should be viewed as hypothesis-generating and requires replication in larger, independent cohorts. Second, as this was a single-centre study, it is difficult to apply the results in the general RA population. Therefore, further multicentre studies would be necessary to confirm the clinical utility of miR-134-5p as biomarker in RA. Third, the discovery RA vs healthy control miRNA-seq signature did not replicate in the RT-qPCR validation for the tested candidates. This may reflect cross-platform differences, cohort heterogeneity, and the limited discovery sample size (with potential effect-size inflation). Although we used standardized pre-analytics, spike-in and haemolysis controls, and reference-miRNA normalization, residual technical variability or batch effects cannot be fully excluded; thus, this diagnostic signature should be considered exploratory and requires replication in larger cohorts. In addition, we did not perform mechanistic *in vitro* validation of predicted miR-134-5p targets (e.g., TP73, MAPK3) in RA synovial fibroblasts or macrophages; such experiments will be addressed in future work to better connect the biomarker signal with relevant inflammatory pathways (including macrophage polarization).Finally, it is important to note that miRNA profiles are compartment-dependent: plasma reflects an integrated systemic signal, whereas PBMC and synovial samples better capture immune-cell and local joint processes. Therefore, diagnostic signatures described in PBMC/synovium may not fully translate to plasma, and cross-platform differences (miRNA-seq vs RT-qPCR) and dilution effects may contribute to limited replication of RA vs HC findings (55). In this context, while miR-223-3p and miR-142-3p are recurrent in previous RA panels (often reflecting inflammatory/myeloid activity) (36–38), our main validated finding was the baseline association of miR-134-5p with anti-TNF non-response, consistent with a predictive rather than pharmacodynamic marker.

## Conclusions

This two−stage study identified a 45−miRNA RA patients vs HC signature and a 9−miRNA baseline signature associated with response to anti−TNF. miR−134−5p was the only candidate validated by RT−qPCR, independently predicting non−response after adjustment for age and sex, supporting further studies addressing its use in pre-treatment stratification. Target mapping (*TP73*, *MAPK3*, *MYC*, *DLX5*) anchors the signature in apoptosis, MAPK/ERK signalling, and cell−cycle control by TNF. Lack of on−treatment modulation indicates predictive rather than short−term pharmacodynamic behaviour. Overall, our results present miR-134-5p as a promising candidate associated with non-response in RA patients. Nevertheless, larger multicentre studies including more diverse clinical profiles are required to validate our results prior its clinical application, and to elucidate the underlying mechanisms of both the miR-134-5p and the 9-miRNA signatures associated with response to anti-TNF treatment.

## Data Availability

The raw sequencing data generated in this study are available in the NCBI Sequence Read Archive (SRA) under BioProject accession number PRJNA1436029 (https://www.ncbi.nlm.nih.gov/bioproject/1436029).

## Supplementary material

Supplementary Table 1 TaqMan probe used for each miRNA. miRNAs considered endogenous are in bold.

Supplementary Table 2 Bowtie2 result.

Supplementary Table 3 Multivariable logistic regression analyses of candidate plasma miRNAs associated with rheumatoid arthritis (RA vs healthy controls) and with anti-TNF response (responders vs non-responders), using expanded adjustment sets. RA, rheumatoid arthritis; HC, healthy controls; miRNA, microRNA; OR, odds ratio; CI, confidence interval; DAS28-CRP, Disease Activity Score in 28 joints using C-reactive protein. *p < 0.05 in the multivariable model. All logistic regression models comparing RA vs healthy controls (HC) were adjusted for age, sex, and smoking status; smoking status was significant in all RA vs HC analyses. Models comparing responders vs non-responders were adjusted for age, sex, smoking status, baseline DAS28-CRP, and baseline glucocorticoid use; smoking status showed a trend toward association in these models (p < 0.15) but did not reach conventional statistical significance (p ≥ 0.05).

Supplementary Table 4 Univariate and multivariable logistic regression of baseline clinical factors and miR-134-5p associated with non-response to anti-TNF therapy in rheumatoid arthritis. * Nagelkerke R²=0.256. anti-TNF, tumour necrosis factor inhibitor; CI, confidence interval; DAS28, Disease Activity Score in 28 joints; OR, odds ratio; RA, rheumatoid arthritis. DAS28-CRP, Disease Activity Score in 28 joints using C-reactive protein.

Supplementary Figure 1 PCA Result.
