# Supplementary figures and images for: Plasma miR-134-5p: a candidate biomarker for predicting non-response to anti-TNF therapy in rheumatoid arthritis
Source: Front Immunol. 2026 Mar 17;17:1783026. doi: 10.3389/fimmu.2026.1783026 (PMC13036163; doi:10.3389/fimmu.2026.1783026)

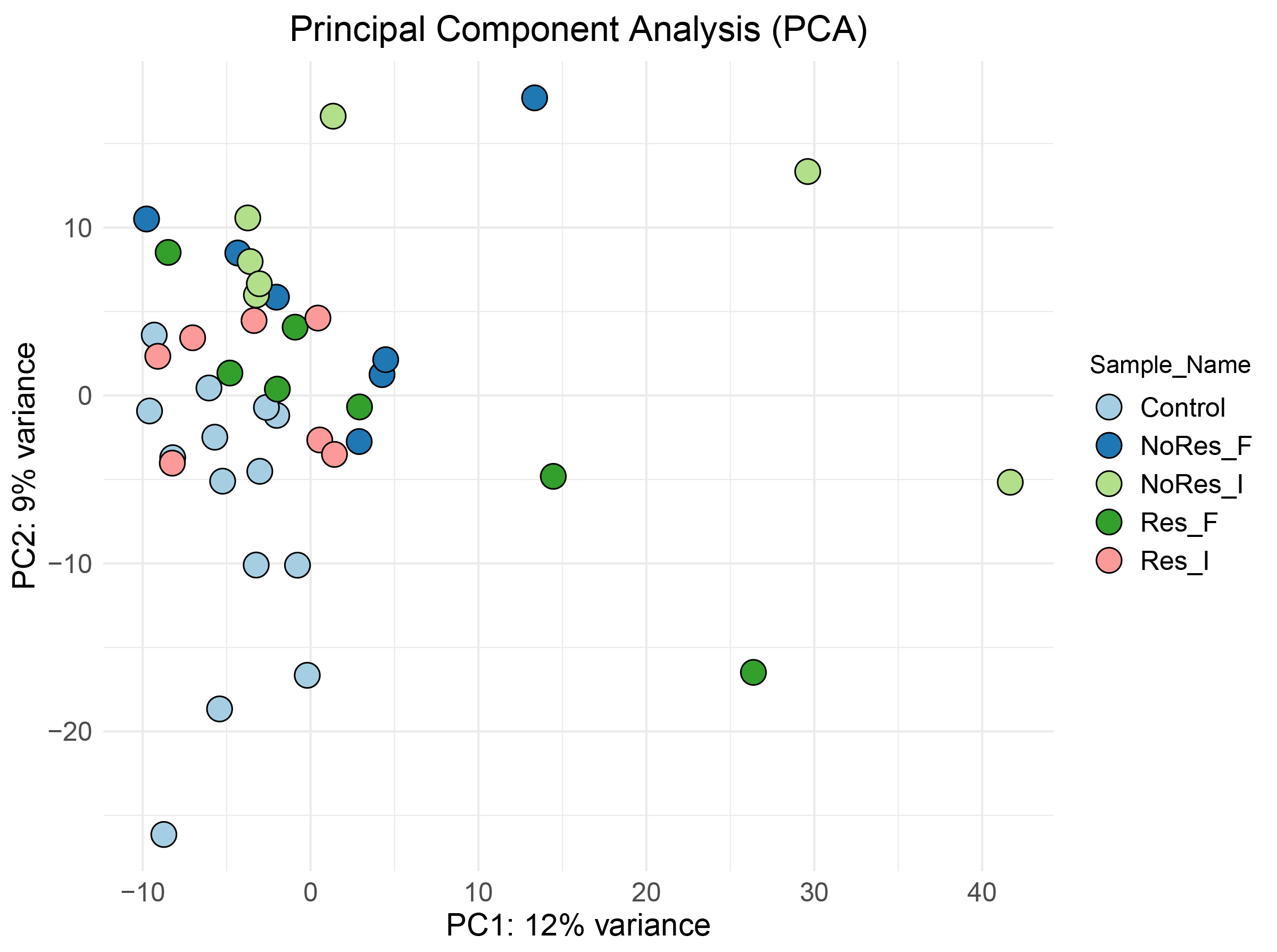

Supplement: Supplementary file 1 [file Image1.jpeg]
