# Supplementary material for: Plasma miR-134-5p: a candidate biomarker for predicting non-response to anti-TNF therapy in rheumatoid arthritis
Source: Front Immunol. 2026 Mar 17;17:1783026. doi: 10.3389/fimmu.2026.1783026 (PMC13036163; doi:10.3389/fimmu.2026.1783026)
Supplement: Supplementary Table 1 — TaqMan probe used for each miRNA. miRNAs considered endogenous are in bold. [file Table1.docx]

| **S. Table 1. TaqMan probe used for each miRNA** | | |
| --- | --- | --- |
| **Assay ID** | **Assay Name** | **Sequence** |
| 478412_mir | hsa-miR-106b-5p | UAAAGUGCUGACAGUGCAGAU |
| 477827_mir | hsa-miR-92a-3p | UAUUGCACUUGUCCCGGCCUGU |
| 477983_mir | hsa-miR-223-3p | UGUCAGUUUGUCAAAUACCCCA |
| 478107_mir | hsa-miR-451a | AAACCGUUACCAUUACUGAGUU |
| 477860_mir | hsa-miR-16-5p | UAGCAGCACGUAAAUAUUGGCG |
| 477910_mir | hsa-miR-142-3p | UGUAGUGUUUCCUACUUUAUGGA |
| 477901_mir | hsa-miR-134-5p | UGUGACUGGUUGACCAGAGGGG |
| **478817_mir** | **hsa-miR-3184-5p** | **UGAGGGGCCUCAGACCGAGCUUUU** |
| **477892_mir** | **hsa-miR-128-3p** | **UCACAGUGAACCGGUCUCUUU** |
| **477848_mir** | **hsa-let-7d-3p** | **CUAUACGACCUGCUGCCUUUCU** |
| miRNAs considered endogenous are in bold. | | |

S. table 2: Bowtie2 result

| **Sample** | **SE mapped uniquely** | **SE multimapped** | **SE not aligned** |
| --- | --- | --- | --- |
| Control_rep10_bowtie | 609956 | 652941 | 10561144 |
| Control_rep11_bowtie | 775412 | 702483 | 8043477 |
| Control_rep12_bowtie | 639404 | 719848 | 6628353 |
| Control_rep14_bowtie | 572271 | 503003 | 5544401 |
| Control_rep15_bowtie | 332859 | 255126 | 6020362 |
| Control_rep1_bowtie | 842563 | 801282 | 10811229 |
| Control_rep2_bowtie | 431461 | 402311 | 3422258 |
| Control_rep3_bowtie | 522156 | 431332 | 5369449 |
| Control_rep4_bowtie | 880365 | 970138 | 11133267 |
| Control_rep5_bowtie | 526576 | 558157 | 7557588 |
| Control_rep6_bowtie | 624350 | 565459 | 6419280 |
| Control_rep7_bowtie | 560751 | 701775 | 7168733 |
| Control_rep8_bowtie | 237684 | 195454 | 3421122 |
| Control_rep9_bowtie | 273690 | 244377 | 4261553 |
| NoRes_F_rep1_bowtie | 446345 | 478312 | 7401829 |
| NoRes_F_rep2_bowtie | 443166 | 357119 | 6933026 |
| NoRes_F_rep3_bowtie | 499973 | 117617 | 4666639 |
| NoRes_F_rep4_bowtie | 675881 | 564145 | 5714798 |
| NoRes_F_rep5_bowtie | 1258543 | 535698 | 7565831 |
| NoRes_F_rep6_bowtie | 777617 | 758109 | 8537298 |
| NoRes_F_rep7_bowtie | 602152 | 487180 | 9181896 |
| NoRes_I_rep1_bowtie | 18583 | 19743 | 1080739 |
| NoRes_I_rep2_bowtie | 411591 | 431124 | 4498286 |
| NoRes_I_rep3_bowtie | 806919 | 1458931 | 7898542 |
| NoRes_I_rep4_bowtie | 67367 | 120588 | 1747000 |
| NoRes_I_rep5_bowtie | 442255 | 244374 | 4795093 |
| NoRes_I_rep6_bowtie | 466878 | 307989 | 5438236 |
| NoRes_I_rep7_bowtie | 537651 | 458210 | 5388854 |
| Res_F_rep1_bowtie | 1198425 | 1415155 | 15913899 |
| Res_F_rep2_bowtie | 432318 | 402548 | 10476430 |
| Res_F_rep3_bowtie | 994005 | 725235 | 14235208 |
| Res_F_rep4_bowtie | 310398 | 355815 | 9829269 |
| Res_F_rep5_bowtie | 1186878 | 1505448 | 10280376 |
| Res_F_rep6_bowtie | 473141 | 255827 | 5293497 |
| Res_F_rep7_bowtie | 270901 | 178228 | 4965635 |
| Res_I_rep1_bowtie | 1279006 | 1504330 | 15174583 |
| Res_I_rep2_bowtie | 1337153 | 1680148 | 11220142 |
| Res_I_rep3_bowtie | 604697 | 508055 | 8625876 |
| Res_I_rep4_bowtie | 825751 | 844931 | 10579443 |
| Res_I_rep5_bowtie | 512509 | 783288 | 11780329 |
| Res_I_rep6_bowtie | 261217 | 183384 | 22687518 |
| Res_I_rep7_bowtie | 659133 | 462184 | 8751529 |

| S. Table 3. Multivariable logistic regression analyses of candidate plasma miRNAs associated with rheumatoid arthritis (RA vs healthy controls) and with anti-TNF response (responders vs non-responders), using expanded adjustment sets. | | |
| --- | --- | --- |
| miRNA | RA patients vs HC | Responders vs non-responders |
| miR-106b-5p | R^2^ = 0.061  OR: 0.982, 95%CI (0.65-1.46)  *p* = 0.931 | R^2^ = 0.074  OR: 1.110, 95%CI (0.71-1.71)  *p* = 0.640 |
| miR-92a-3p | R^2^ = 0.060  OR: 1.116, 95%CI (0.67-1.85)  *p* = 0.672 | R^2^ = 0.077  OR: 1.193, 95%CI (0.62-1.90)  *p* = 0.753 |
| miR-223-3p | R^2^ = 0.065  OR: 0.952, 95%CI (0.61-1.46)  *p* = 0.822 | R^2^ = 0.076  OR: 1.039, 95%CI (0.64-1.66)  *p* = 0.873 |
| miR-451-a | R^2^ = 0.061  OR: 1.063, 95%CI (0.98-1.13)  *p* = 0.562 | R^2^ = 0.074  OR: 0.942, 95%CI (0.64-1.36)  *p* = 0.754 |
| miR-16-5p | R^2^ = 0.067  OR: 1.123, 95%CI (0.76-1.64)  *p* = 0.554 | R^2^ = 0.080  OR: 0.969, 95%CI (0.62-1.50)  *p* = 0.886 |
| miR-142-3p | R^2^ = 0.065  OR: 0.959, 95%CI (0.72-1.27)  *p* = 0.773 | R^2^ = 0.085  OR: 1.118, 95%CI (0.82-1.51)  *p* = 0.471 |
| miR-134-5p | R^2^ = 0.067  OR: 0.847, 95%CI (0.59-1.20)  *p* = 0.351 | R^2^ = 0.256  OR: 1.641 (1.002-2.677)  ***p* = 0.043*** |
| Abbreviations: RA, rheumatoid arthritis; HC, healthy controls; miRNA, microRNA; OR, odds ratio; CI, confidence interval; DAS28-CRP, Disease Activity Score in 28 joints using C-reactive protein. *p < 0.05 in the multivariable model. All logistic regression models comparing RA vs healthy controls (HC) were adjusted for age, sex, and smoking status; smoking status was significant in all RA vs HC analyses. Models comparing responders vs non-responders were adjusted for age, sex, smoking status, baseline DAS28-CRP, and baseline glucocorticoid use; smoking status showed a trend toward association in these models (p < 0.15) but did not reach conventional statistical significance (p ≥ 0.05). | | |

S. Table 4. *Univariate and multivariable logistic regression of baseline clinical factors and miR-134-5p associated with non-response to anti-TNF therapy in rheumatoid arthritis.*

| Responders vs non-responders | | | |
| --- | --- | --- | --- |
| Variable | Univariate OR (95%CI) | Multivariate OR  (95%CI) | p-value |
| Age, years | 1.008 (0.968-1.038) |  |  |
| Female sex | 0.681 (0.202-2.293) |  |  |
| Smoking habit | 2.731 (0.984-7.578) |  |  |
| Average DAS28 | 1.011 (0.649-1.577) |  |  |
| Glucocorticoids | 1.355 (0.440-4.176) |  |  |
| miR-134-5p | 1.640 (1.003-2.680) | 1.641 (1.002-2.677) | 0.043 |

*** *Nagelkerke R²=0.256*

*Abbreviations: anti-TNF, tumour necrosis factor inhibitor; CI, confidence interval; DAS28, Disease Activity Score in 28 joints; OR, odds ratio; RA, rheumatoid arthritis. DAS28-CRP, Disease Activity Score in 28 joints using C-reactive protein.*
